# Approximate reinforcement learning to control beaconing congestion in distributed networks

**DOI:** 10.1038/s41598-021-04123-9

**Published:** 2022-01-07

**Authors:** J. Aznar-Poveda, A.-J. García-Sánchez, E. Egea-López, J. García-Haro

**Affiliations:** Department of Information and Communications Technologies, Universidad Politécnica de Cartagena, 30202 Cartagena, Spain

**Keywords:** Electrical and electronic engineering, Computer science, Information technology, Software

## Abstract

In vehicular communications, the increase of the channel load caused by excessive periodical messages (beacons) is an important aspect which must be controlled to ensure the appropriate operation of safety applications and driver-assistance systems. To date, the majority of congestion control solutions involve including additional information in the payload of the messages transmitted, which may jeopardize the appropriate operation of these control solutions when channel conditions are unfavorable, provoking packet losses. This study exploits the advantages of non-cooperative, distributed beaconing allocation, in which vehicles operate independently without requiring any costly road infrastructure. In particular, we formulate the beaconing rate control problem as a Markov Decision Process and solve it using approximate reinforcement learning to carry out optimal actions. Results obtained were compared with other traditional solutions, revealing that our approach, called SSFA, is able to keep a certain fraction of the channel capacity available, which guarantees the delivery of emergency-related notifications with faster convergence than other proposals. Moreover, good performance was obtained in terms of packet delivery and collision ratios.

## Introduction

Transportation is evolving in increasingly overpopulated cities due to a growing demand for goods and human transit. Unless current trends change, the number of vehicles on the road is predicted to triple by 2050^1^, which will also contribute to increasing vehicle-related crashes, injuries, and death ratios. Under these circumstances, Intelligent Transportation Systems (ITS) and Vehicle-to-Vehicle (V2V) communications are expected to reduce these detrimental effects and extend the capabilities of numerous driver-assistance systems and driverless vehicles^2^. The European Telecommunications Standards Institute (ETSI) defines V2V communications over the ITS-G5 radio channel, a 10 MHz control channel at the 5.9 GHz band of the IEEE 802.11p standard^3^. The ETSI Cooperative Awareness Service (CAS)^4^ transmits periodic broadcast single-hop messages, called beacons, throughout this control channel (Channel 172 in the US). Formally called Cooperative Awareness Messages (CAM) in Europe or Basic Safety Messages (BSM) in the US, beacons are responsible for disseminating status and environmental information among vehicles, where specific information such as position, speed, acceleration, direction, or vehicle dimension are employed to track and predict vehicle behavior. This broadcast information supports many safety applications and is crucial to reducing the risk of collision among vehicles or other undesired events^5–7^.

As the number of CAS beacons increases, the aggregated load can easily saturate the communication channel, compromising packet reception, and therefore endangering vehicle situation awareness. In this scenario, many safety applications^5–7^ based on beacons could receive outdated and inaccurate information. Furthermore, congestion negatively affects other services transmitted over the ITS-G5 radio channel, like Decentralized Environmental Notification (DEN), which notifies emergency services whenever an accident occurs on the road^8^. Not receiving such event-related messages, called DEN Messages (DENM), is of critical importance to the safety and health of road users (drivers, passengers, and pedestrians). To guarantee the delivery of these emergency-related messages (DENM), as well as to provide enough cooperation awareness (using CAM messages) to satisfy safety application requirements, a certain fraction of the channel capacity should be available. The upper limit of the channel load that can be dedicated to beaconing is usually called Maximum Beaconing Load (MBL). To satisfy the MBL constraint, the ETSI standard defines a Cross-Layer Decentralized Congestion Control (DCC) Management Entity^9^ to prevent the ITS-G5 radio channel from overloading.

Several transmission parameters can be tuned over time according to channel requirements and congestion. Keeping this in mind, the most widespread mechanism used to restrict congestion is decreasing the number of beacons transmitted per second. In^10^, authors proposed the LIMERIC method, in which each vehicle linearly updates its own rate (metric) depending on the total channel load, thus orienting rates toward a certain target value. This algorithm became so extended that even the standardization process included it in the DCC mechanism^9^. PULSAR^11^ was another popular rate-based control algorithm using Additive Increase Multiplicative Decrease (AIMD) with feedback from 2-hop neighbors. Since the convergence of LIMERIC has not been tested when some vehicles are out of range with each other, PULSAR was used in combination with LIMERIC to solve this issue^12^. With this combination, every vehicle sharing a link converges to the beaconing rate employed by the most congested link. The disadvantage of this solution is that it might unnecessarily decrease the beaconing rate of some vehicles, even though they are not congested. In^13^, authors proposed FABRIC, which dealt with congestion control as a Network Utilization Maximization (NUM) problem^14,15^ to optimally allocate beaconing rates. This approach allowed the design of simple algorithms with proven convergence. Note that these previous solutions are pure congestion controls in which only channel load is used to update beaconing rates. Conversely, other approaches found in the literature cope with the beaconing rate metric from some kind of prioritization. For instance, beacon inter-reception time is employed in^16^ to improve vehicle awareness. Similarly, other works adjust the beaconing rate in an attempt to minimize position tracking error with respect to other vehicles^17^. This was actually included in the US DCC standard^18^. Under this umbrella, the work in^19^, called EMBARC, is based on LIMERIC but integrates the tracking error algorithm of^20^. Several works define some risk metrics related to vehicle dynamics and traffic situations, such as^21–24^. In^21^, collision probability was employed to adjust the message or beaconing rate at intersections. The works^22,23^ employed tailgating collision risk to assign more resources to more dangerous vehicles. TTCC^24^ extended FABRIC to transmit beacons using a more generic time-to-collision metric. Vehicle density was also used to fit beacon generation over time^25,26^.

Most of the approaches mentioned above involve including additional information in beacon payloads. This implies that the congestion control procedure depends on beacon reception, which may disrupt congestion control performance in unfavorable channel conditions. As suggested in^27^, tracking errors should be included in the congestion alleviation mechanisms. However, there is a more straightforward solution: not relying on neighbors' information to control congestion. These kinds of algorithms, commonly known as non-cooperative, are able to obtain a global change by means of individual actions. The most representative one is NORAC^28^, a distributed beaconing rate control that employs game theory as its optimization core. As expected, NORAC does not involve exchanging control information, so each vehicle independently fits its beaconing rate according to the channel load measured. Despite the fact that some parameters can be used to adjust the behavior of NORAC, the MBL cannot be explicitly set. This leads to an insufficient or excessive channel load unless an appropriate combination of parameters is selected. However, non-cooperative proposals provide simple but effective resource allocation with very low computational cost, which results in a faster convergence speed to appropriate beaconing rates.

In this paper, we thoroughly discuss all these aforementioned aspects and conceive novel, non-cooperative congestion control capable of attaining an optimal MBL. Similar to game theory, we explore how decision theory and novel reinforcement learning (RL) techniques^29^ can be applied to resolve a distributed optimization problem. As far as we know, most of the RL-based works require some kind of infrastructure; that is, they are designed for cellular networks^30–32^ or employ a more complex combination of parameters^31,33–36^. However, none of them introduce simple, reliable, and fast beaconing rate control to alleviate congestion for V2V communications. We make use of a finite Markov Decision Problem (MDP) to formulate both the road environment and congestion control, which is later solved using approximate solution methods. In particular, we apply on-policy control with function approximation, which, unlike tabular solutions, allows us to generalize previous states to derive sensible decisions when new states are encountered. The resulting parameterized model can be applied by vehicles so the most appropriate beaconing rate is arrived at very efficiently in terms of runtime and computational cost, which is of great importance in congested scenarios. Results show that the policy, together with the model evaluated, called SSFA, successfully adjusts the channel load to an appropriate level. This means that road safety services, such as DEN, maintain a certain reserved bandwidth to guarantee the delivery of DENM notifications. Also, the proposed congestion alleviation mechanism does not require the installation of any costly infrastructure on the road (distributed) and does not depend on channel conditions to work properly (non-cooperative).

The remainder of this article is organized as follows. In “MDP formulation for congestion alleviation” section, we describe the resource (beaconing rate) allocation problem of V2V communications more thoroughly and introduce our proposal. Then, we validate it in “Results” section, comparing it with other algorithms and discussing the obtained results. Finally, “Conclusion” section summarizes the main conclusions.

## MDP formulation for congestion alleviation

Excessive channel load might increase packet loss and hamper the operation of safety applications with outdated information, not to mention the fatal consequences of not receiving emergency notifications or DENMs. To overcome this problem, congestion control maintains the channel load near a certain target value, defined as the Maximum Beaconing Load (MBL). According to several works^13,28,37,38^, the MBL is assumed to be around 60 or 70 percent of channel capacity (C), leaving the remaining percentage of the channel free to guarantee the delivery of DEN-related messages and other essential services. Since no a priori information or data about the (road) environment is available, we model the beaconing rate allocation problem as a finite Markov Decision Process (MDP), which is the basis of Reinforcement Learning (RL), to optimally satisfy this MBL constraint using discrete actions. In such a way, each vehicle takes actions, performs transitions among different states, and obtains different rewards depending on how well congestion is alleviated. This will be solved by means of approximated reinforcement learning techniques. The parameterized model resulting from these learning techniques can be easily evaluated by vehicles, causing the algorithm to converge significantly faster than other approaches^13,28^.

MDPs are often employed to formulate optimization problems and later solve them by deriving optimal sequences of actions. This is particularly appropriate for complex environments that are partially random and difficult to predict. MDPs are mainly comprised of several entities. Firstly, *agents* are the learner entities that continuously seek for optimal behavior. In our case, vehicles evaluate policies to keep channel congestion under control. Secondly, the *environment* (road) is everything outside the agent (pedestrians, roads, or other agents) able to alter the agent *state*. The external environmental situation and the internal agent conditions are called *state*, usually defined as a vector $s\in \mathcal{S}$, with $\mathcal{S}$ being the set of possible states. The agent is able to vary its state, from *s* to *s'*, by carrying out actions $a\in \mathcal{A}\left(s\right).$ Every time this happens, the environment is modified, and the agent obtains a *reward r* according to how appropriate the behavior of the agent has been. The agent acts over time in a bid to maximize the reward obtained, which can be modeled as a function of the state *s* and the action taken *a*, i.e., $r(s,a)=f\left(s,a\right)\in {\mathbb{R}}$.

The relationships among the different entities are usually determined by state-transition models, depicted by probabilities of transitioning among states. Nonetheless, in realistic scenarios with fast variations and partial information, MDP-solving algorithms employ a mapping between states and actions called policy; that is $\pi :\mathcal{S}\to \mathcal{A}$. Consequently, the main goal of the MDP-solving algorithm is to reach the optimal policy ${\pi }^{*}$ that maximizes the accumulated sum of rewards during the entire training of the agent.

### Particularization of actions and states

As mentioned above, the agents of the proposed MDP model, represented by vehicles, sense their environment to adequately adjust their beaconing rate, and thus reduce overall channel congestion. These changes are called *actions*, and they allow vehicles to reduce, maintain, or increase their current beaconing rate within the limits stated in the standard (1–10 Hz) ^4^. The set of available discrete actions is called action space and is crucial to obtain a good training efficiency and later algorithm accuracy. For instance, too small actions (e.g. < 0.1 Hz) may lead to a more accurate solution but involving a huge state space, which takes much longer to be trained without incurring inaccuracies since many states may never be visited after a while. Once deployed, the convergence of our algorithm could also be affected if too many steps are required. In contrast, too big actions (e.g. > 1 Hz) simplify the training of the model at the expense of accuracy, so that the optimal value would rarely be reached over time. Note that the number of available actions can also make the dimension of the state space grow exponentially, to the detriment of effective training process. Therefore, an appropriate balance should be struck between training efficiency (directly related to the size of the state space) and proximity to the optimal value, in order to appropriately select the set of actions. In our particular case, the action space $\mathcal{A}\left(s\right)=\{0,\pm 0.5\}$ Hz was used.

Congestion is usually measured by using the Channel Busy Ratio (CBR), defined as the fraction of time (typically 1 s) during which the channel is busy due to transmissions or receptions. Another way of understanding the CBR is as the fraction of the channel load (sum of the neighbors’ beaconing rates), over channel capacity. Note that this metric reflects external environment conditions. For instance, given a beaconing rate, a low measured CBR may be due (i) to a channel with high fading (lost packets results in a lower measured CBR) or (ii) because of having few neighbors. From the point of view of our solution, the action to perform would be the same irrespective of the real cause. In that sense, the CBR captures well the particularities of different scenarios. Because of this, the performance of our proposed solution is robust to variations in the channel model or radio propagation effects, as will be shown in the results section.

**Figure 1:**
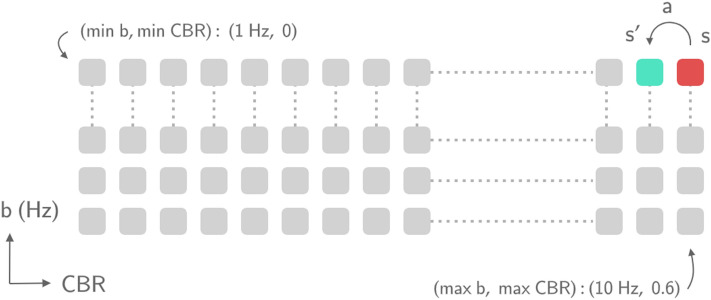
Two-dimensional space of states employed to model the beaconing rate allocation problem as an MDP. Axes represent each constituent element of the available states of the MDP: beaconing rate and CBR.

Taking this into account, let us define the states of the MDP model as the tuple comprised of the current beaconing rate and the CBR measured $s=(b, CBR)$. Up to 789 different CBR values (60% of the channel capacity in beacons per second) from 0 to 0.6 (MBL/C) are included in the MDP model, which results in 15,780 different tuple states. Every sensed state above or below these limits would result in a decrease or increase of the beaconing rate, respectively. As shown in Fig. 1, the proposed space of states can be illustrated on a two-dimensional plane, where the axes represent both the current beaconing rate and the CBR measured. When executing an action $a\in \mathcal{A}\left(s\right)$, the environment gives a new state *s’* back to the vehicle. The beaconing rate only applies the action value to the state. If, for instance, a lot of vehicles are transmitting at 10 Hz (beaconing rate) but suddenly they experience slight congestion and *a* = -0.5, they will decrease their beaconing rate to 9.5 Hz.

### Reward function

With each transition among states, the agent obtains a reward $r\left(s,a\right)\in {\mathbb{R}}$, which denotes how much the current behavior differs from the desired behavior. In other words, maximizing the accumulated reward allows the agent to approach the optimal transmission parameters recommended by the optimal policy ${\pi }^{*}$. In our case, the desired behavior is to maintain the channel load around the MBL, typically between 60 and 70 percent of channel capacity. Note that higher loads may increase packet loss, jeopardizing vehicle context awareness and preventing emergency-related messages from being decoded. In contrast, a lower CBR would imply that the channel is underused, causing a loss of awareness. To overcome these drawbacks and thus achieve an optimal channel load, the reward is shaped according to the following function:

1 $$r\left(x\right)=-x sign\left(x-\delta \right),$$

where *sign* is the signum function shifted by target value *δ*. In our case, the input *x* is the CBR, whereas the target value *δ* would be the MBL over channel capacity. Let us denote this upper CBR limit as the Maximum Beaconing Ratio ($MBR=MBL/C$). With this function, an increasing positive reward is obtained as the CBR approaches the target value MBR (0.6–0.7). However, if the CBR overruns this limit, a decreasing negative reward is obtained. These negative rewards speed up the learning process compared to using only positive ones, as proved in^29^. In short, reaching the MBR limit not only allows us to reduce congestion and leave a certain fraction of the channel free to guarantee the delivery of emergency-related messages but also prevents channel underutilization.

### Policy derivation

Once the states, actions, and rewards of the MDP have been defined, agents should learn the most advantageous policy; that is, to determine the sequence of actions for which the total reward is maximized. To this end, we employ **S**emi-gradient **S**ARSA with **F**unction **A**pproximation (hence the name of our congestion alleviation mechanism: SSFA). SARSA iteratively updates the policy to achieve as large a reward as possible over time. Its name comes from the fact that the action is updated in function of the current state ($s$), the action selected ($a$), the reward obtained ($r$), the new state (${s}^{^{\prime}}$), and the next action selected by the agent ($a^{\prime}$) in the new state. In difference to the training environment described above which was defined with discrete MDP states, the state space is continuous in real conditions (or their simulated counterparts) while evaluating the policy. Making sensible decisions in these unknown states entails generalizing from previous states that are similar to the current one. To this end, we attempt to generalize using function approximation; that is, we approximate the state-action value function, $Q\left(s,a\right)$, as a parameterized function $\widehat{Q}\left(s,a\right)$, as follows:

2 $$Q\left(s,a\right)\approx \widehat{Q}\left(s,a\right)={\theta }_{0}+{\theta }_{1}{x}_{1}\left(s,a\right)+\dots +{\theta }_{n}{x}_{n}\left(s, a\right),$$

where $\theta \in {\mathbb{R}}^{n}$ is an n-dimensional weight vector to be learned and $\overline{x }=\langle {x}_{1},\dots ,{x}_{n}\rangle$ the n-tuple (n = 5) comprised of the following features:

- ${x}_{1}\left(s,a\right)$ represents an additional reward given whenever ideal behavior is reached. Therefore, it has a value of 1 if the vehicle senses a CBR = MBR and 0 otherwise.
- ${x}_{2}(s,a)$ and ${x}_{3}(s,a)$ are congestion indicators, which are useful to lead the vehicle to the desired behavior and to define whether the channel load is congested or not. They have a value of 1 if the vehicle has high or low congestion, respectively; that is, the CBR experienced is above or below the MBR, and 0 otherwise.
- ${x}_{4}(s,a)$ and ${x}_{5}(s,a)$ provide subtle information about how the algorithm should proceed in detail. They assess whether the associated action is approaching or moving away from the desired behavior. In particular, they have a value of 1 if the CBR measured after carrying out the action is closer or further from the MBR, respectively, and 0 otherwise.

As can be observed, these five functions are modeled to obtain the desired behavior so the algorithm does not depend so much on how the reward is shaped. In short, using function approximation not only allows better generalization when assessing the policy but also speeds up the learning process and eases reward tuning. It is worth noting that more sophisticated RL algorithms, suitable for continuous action spaces, could be used to directly predict the optimal beaconing rate. Nevertheless, these solutions entail tough reward modeling to obtain the desired behavior in the right way as well as longer training times and subtle hyperparameter tuning. In contrast, we provide a simple congestion alleviation mechanism that can be trained straightforwardly and is ready to be deployed in realistic scenarios. Furthermore, as will be seen in the next section, the results obtained are close to the optimal values proposed by baseline works, and more complex algorithms might not mean a significant improvement.

The complete environment and the solving algorithm of the MDP model proposed have been implemented in Python, using different classes, objects, and advanced libraries, like NumPy^39^, to obtain efficient data processing. The environment is represented by a set of vehicles arbitrarily located on a two-dimensional plane, as would occur on realistic roads. The interactions between the agents and the environment, such as rewards and transitions among states, are also implemented. In this (training) environment, each vehicle includes its current state (CBR and beaconing rate), transmission power, spatial location, and the set of allowed actions. Note that the proposed congestion control algorithm is not influenced by channel model or propagation effects so we assume here a free-space channel model and sufficient transmission power to permit vehicles to be in coverage with each other. In this way, numerous congestion levels represented by different numbers of vehicles allow a policy able to respond quickly to each CBR measured, from 0 to 0.6, to be obtained. This CBR is controlled in a distributed fashion by each vehicle according to the way the reward is modeled.

The semi-gradient SARSA with function approximations described in Algorithm 1 was implemented in a different class from the environment. Firstly, the parameterized action-state pairs, or $\widehat{Q}$-values, are initialized to zero. For each episode, the environment generates as many vehicles as required to represent the CBR indicated in the current state and iteratively calculates the expected rewards and updated $\widehat{Q}$-values. For instance, if the initial state is $s=\left(b,CBR\right)= (10 Hz, 0.5)$, the environment will create as many vehicles transmitting at 10 Hz as possible to obtain a CBR = 0.5. The algorithm will recommend actions to every vehicle equally until reaching the optimal policy ${\pi }^{*} = f({\theta }^{*})$ and maximizing the accumulated reward during the training. Note that the policy is shared among vehicles and that overall channel congestion is successfully controlled in this training scenario. As will be shown in the following section, this shared policy will work appropriately even when all the vehicles are not in range of each other since individual contributions lead to the right overall channel load. This is the advantage of non-cooperative algorithms: they can obtain a global change by means of individual actions.
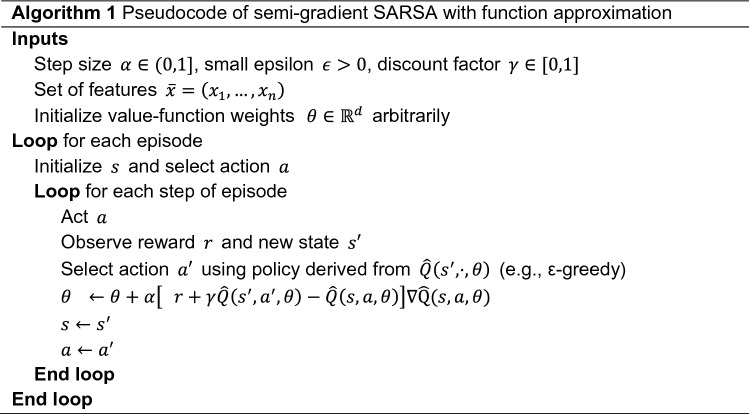


**Figure 2:**
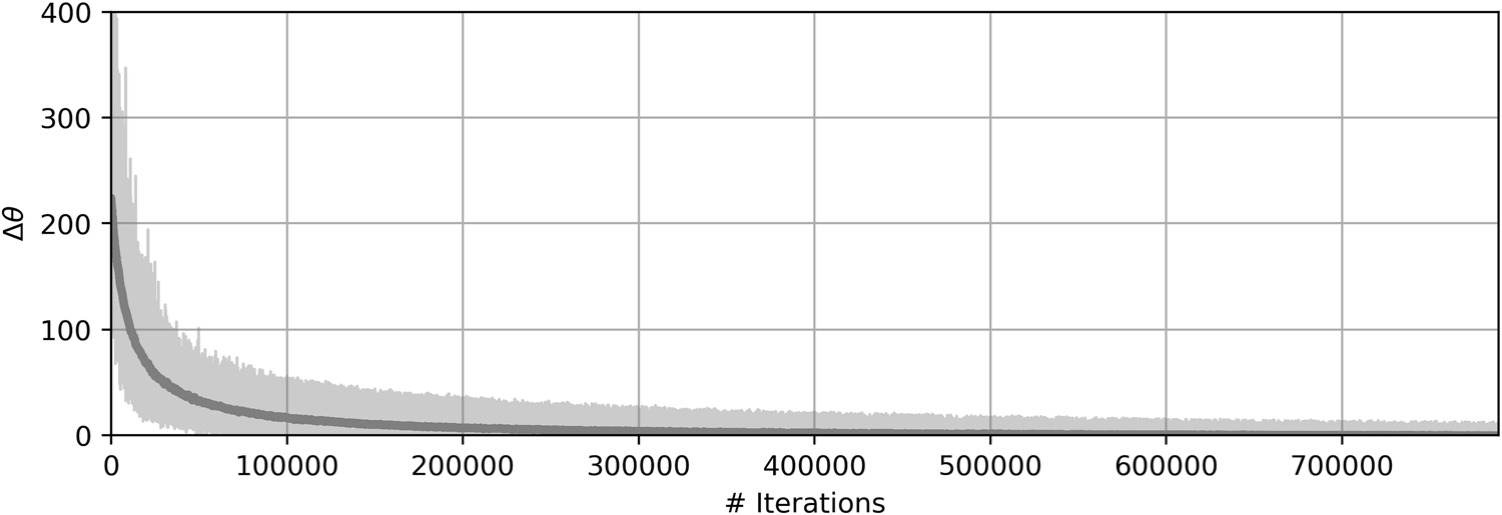
Biggest variation of consecutive $\theta$ values for each episode.

It should be noted that attaining the optimal policy is not guaranteed. For this reason, we continued training until we achieved the desired behavior ($CBR\approx MBR$). To illustrate this, the learning curve of the proposed algorithm has been plotted in Fig. 2 through the biggest change of consecutive $\theta$ vectors, called $\Delta \theta$. This value was calculated as the sum of the difference between the elements of successive $\theta$ vectors. As can be observed, the biggest changes between consecutive $\theta$ values decrease as training moves forward, which implies better performance.

The most meaningful parameters of the environment as well as those employed in the MDP-solving algorithm have been summarized in Table 1. In the next section, the performance of the proposed congestion alleviation mechanism in different motorway and urban scenarios will be thoroughly assessed.

**Table 1:** Training parameters and their values.

Parameter	Value
Discount factor ($\gamma$)	0.9
Step size ($\alpha$)	0.1
Epsilon-greedy probability ($\epsilon$)	0.1
Channel capacity (C)	1315.78 beacons/s
Maximum Beaconing Load (MBL)	789.47 beacons/s
Maximum Beaconing Ratio (MBR)	0.6
Transmission power	500 mW (27 dBm)
Min., Max. beaconing rate	1, 10 Hz
Number of available actions ($|\mathcal{A}|$)	3
Number of available rates ($|\mathcal{B}|$)	20
Number of available CBRs ($|\mathcal{L}|$)	789
Total number of states ($|\mathcal{S}|)$	$|\mathcal{B}|\times |\mathcal{L}|$
Episodes	$(\left|\mathcal{S}\right|\times |\mathcal{A}|)/MBR$
Steps of episode	100

## Results

In this section, a well-trained SSFA model is evaluated using different studies ranging from simple, theoretical evaluations to more complex and realistic simulations in urban and motorway scenarios. To this end, the resulting policy is loaded onto vehicles to execute the SSFA mechanism, as shown in Algorithm 2. Firstly, each vehicle measures the CBR and initializes the beaconing rate (10 Hz by default). Once the vehicles become aware of their state, the policy function modeled by the weights gives the optimal action back. Then, the policy is evaluated as many times as there are different available rates (|$\mathcal{B}$|) as a preemptive measure to avoid overlooking possible inaccuracies in the trained policy. This way, vehicles are led to reduce overall congestion in a distributed and non-cooperative fashion. Note that this is achieved thanks to the individual contributions of all the vehicles in the network, which follow the same policy and act equally under similar states. Finally, according to the received action, SSFA (each vehicle) adjusts the beaconing rate that will be used until the next update.
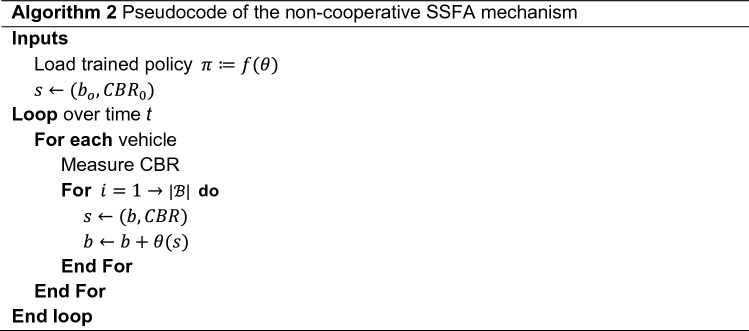


SSFA allocates beaconing rates without cooperation among vehicles and without relying on any base station or road infrastructure. Therefore, we compare it with two similar congestion control mechanisms found in the literature. The first solution in the comparison is NORAC^28^, which employs game theory to allocate the beaconing rate depending on the measured CBR. Nevertheless, as discussed in “Introduction” section, no channel load limit can be explicitly set. In other words, the proper combination of parameters to reach a given MBL is, a priori, unknown, which means that the MBL constraint might not be met when traffic conditions vary. The second comparative solution is FABRIC^13^, which approaches the beaconing rate allocation as a Network Utilization Maximization (NUM) problem with proven convergence. Despite providing optimal allocation, FABRIC entails including Lagrange multipliers in the header of the transmitted messages (these multipliers, also known as prices, $\pi$, should not be confused with the policy that defines agent behavior). This means that vehicles require additional information about their neighbors, which may increase the convergence time. It is noteworthy that the authors of NORAC criticized FABRIC for piggybacking these prices. Nonetheless, we consider that adding a few extra bytes in the heading is not as serious a problem as longer convergence time. The comparison of (i) our non-cooperative approach based on decision theory (RL), (ii) FABRIC, which is a cooperative solution employing NUM, and (iii) NORAC, a non-cooperative solution based on game theory, is performed by making use of the following metrics:

- Channel Busy Ratio (CBR). The CBR is defined as the ratio between channel load and channel capacity. Furthermore, it can be interpreted as the fraction of busy time (typically 1 s) due to transmissions or receptions. As seen throughout this work, this metric represents how much of the channel is used (congested) so it is closely related to packet loss.
- Neighboring vehicles. Finally, together with the CBR, the number of neighbors detected provides valuable insight into the distribution of resources (and context awareness) among vehicles, which should also be considered when assessing the aforementioned algorithms.
- Packet Delivery Ratio (PDR). The PDR is usually defined as the sum of successfully decoded packets with respect to the number of packets transmitted in the network^40,41^. In our particular case, we employ a transmitter-centric approach in which the PDR is measured as the transmitted packets that are successfully received at a certain distance over the total number of packets transmitted. More to the point, the PDR is calculated every 50 m from the source vehicle.
- Packet Collision Ratio (PCR). We assume the PCR as the number of packets lost due to a collision (${n}_{c}$) between the packets successfully decoded (${n}_{s}$) and those lost due to a collision, ${n}_{c}/({n}_{c}+{n}_{s})$, as suggested in^36^.

The scenarios simulated below are not only conducted using different channel conditions and environments, but also a variable number of vehicles. In the following sections, we simulate 650 static vehicles under training conditions, 400 static vehicles under realistic conditions, and up to 400 moving vehicles gradually introduced in a realistic urban scenario. For all these scenarios, a beacon size of 536 bytes and a fixed data rate of 6 Mbps were employed. According to the standard^3^, this results in a total PHY packet duration of 760 $\mu s$ and channel capacity of C = 1315.78 beacons per second. The whole set of simulation parameters are depicted in Table 2.

**Table 2:** OMNeT +  + simulation parameters.

Parameter	Value
Frequency band	5.9 GHz
Channel model	Nakagami-m
Carrier sense threshold	− 92 dBm
Noise floor	− 110 dBm
SNIR threshold	4 dB
Data rate	6 Mbps
Transmission power	500 mW (27 dBm)
Beacon size	4288 bits
Channel capacity (C)	1315.78 beacons/s
Maximum beaconing load (MBL)	789.47 beacons/s
Maximum beaconing ratio (MBR)	0.6
Min., Max. beaconing rate	1, 10 Hz
**FABRIC parameter**	
α	1
β	2.8e−7
ω	1
π_0_	0.001252
**NORAC parameter**	
u_i_	5
pc_i_	0.2

### Evaluation under training conditions

**Figure 3:**
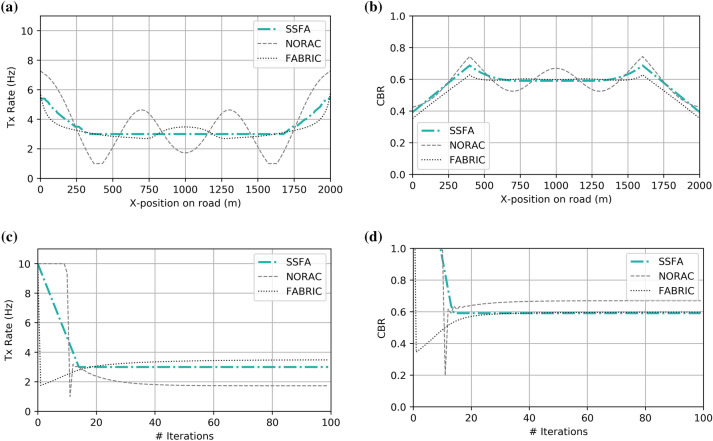
Theoretical comparison (implemented in Python) of the proposed congestion control approach with FABRIC and NORAC. (**a**) Recommended beaconing rate and (**b**) CBR measured for a row of vehicles versus their position on the road; (**c**) Evolution of the beaconing rate and (**d**) CBR of a vehicle located in the middle area of the road over time.

As a first step, we evaluate the proposed congestion control using the Python environment, maintaining most of the training conditions. Therefore, resource allocation is performed theoretically, without sending messages or considering any interference phenomena, headers, or further MAC or PHY protocols. However, unlike the training, in which vehicles were randomly distributed and transmission power was high enough to reach every other vehicle, we now introduce a limited communication range of 400 m. We employ a single row of 650 vehicles evenly spaced along 2000 m. Every algorithm under comparison was run for 100 successive iterations. This evaluation is useful to check whether our proposed mechanism obtains the desired overall congestion (CBR) even when vehicles act in a non-cooperative way. As shown in Fig. 3, the beaconing rate obtained by evaluating the resulting policy (SSFA) is similar to the optimal response of FABRIC. NORAC has a rougher, oscillating shape, although the allocation tends toward the same limit as SSFA and FABRIC by the middle of the row of vehicles. Concerning the CBR, the three algorithms perform well in terms of reaching the MBR constraint, which is satisfied except in the transition to the edges, around 350 and 1600 m. The evolution over time for a vehicle located in the middle of the row was also obtained to study the convergence time. In this case, our proposed SSFA algorithm converges faster (around 15 iterations) than NORAC (40 iterations) and FABRIC (30 iterations).

### Realistic uniformly spaced vehicles

**Figure 4:**
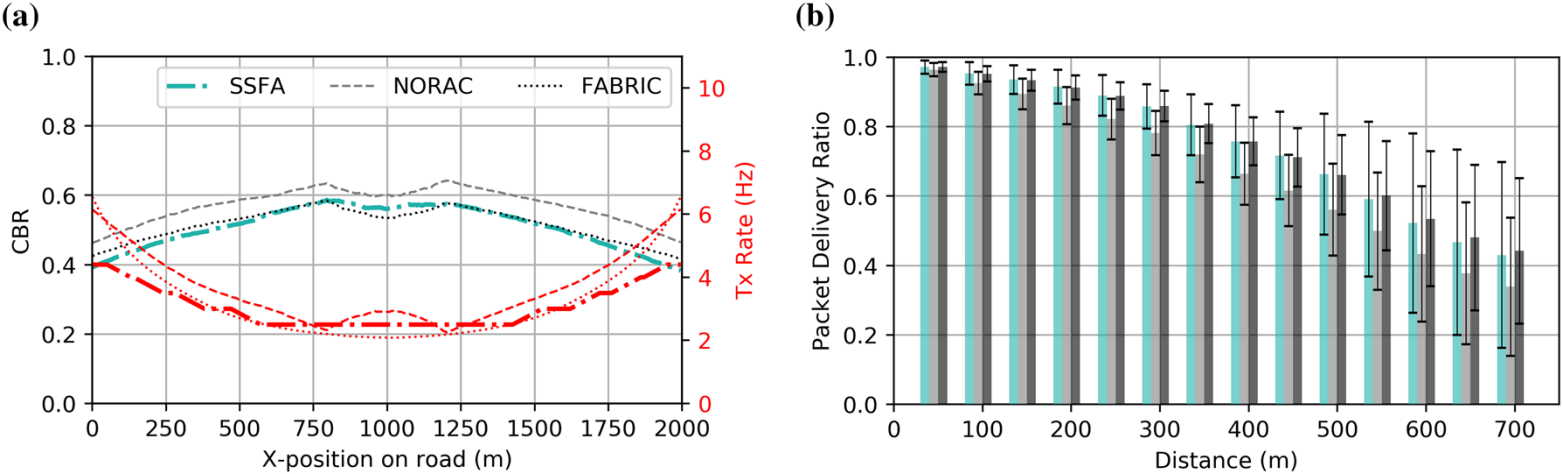
Realistic simulation (OMNeT ++) of our proposed congestion control approach compared to FABRIC and NORAC for an evenly spaced row of vehicles. (**a**) Beaconing rate and CBR measured versus the vehicles' position on the road; (**b**) Packet Delivery Ratio over different distances.

In this subsection, we rigorously assess the performance of each comparative algorithm with the well-known discrete event simulator of networks OMNeT +  + 5.3^42^. The INET 3.5 library^43^ was used to implement the IEEE 802.11p standard as well as realistic channel, propagation, and interference models. To observe whether these realistic conditions affect the process of how resources are allocated, we deploy a similar scenario to the previous subsection. In particular, a row of 400 static vehicles uniformly spaced along 2000 m is simulated for 30 s. As illustrated in Fig. 4, SSFA obtains reliable beaconing rate allocation, comparable to the optimal rate proposed by FABRIC, and similar also to NORAC. Regarding the CBR measured, SSFA and FABRIC provide identical responses, whereas NORAC slightly exceeds the MBR limit. Recall that NORAC does not allow the upper CBR limit to be specified. Therefore, not reaching the desired CBR of 0.6 exactly means that the parameters selected were probably not optimal. This non-compliance brings interesting outcomes in terms of packet delivery ratio, decoded packets, and packet collision ratio, as shown in Fig. 4b and Table 3, respectively. As expected, SSFA and FABRIC reach a similar packet delivery ratio, but that of NORAC is reduced. Such an effect highlights the importance of congestion control and proves that the MBR used (0.6) is the value which allows us to obtain the best performance, as studied in numerous works^13,28,37,38^. Since NORAC surpasses the MBR limit, the beaconing rate, and thus the number of decoded packets, is slightly higher than with the FABRIC and SSFA algorithms. However, the packet collision ratio is significantly higher than in the SSFA and FABRIC approaches. So far, not only does our proposal improve the convergence time but it also obtains excellent results in terms of PDR and PCR. Moreover, it is important to note that, unlike FABRIC, which is based on exchanging prices among neighboring vehicles, SSFA does not depend on channel conditions or packet delivery to operate properly.

**Table 3:** Packet Collision Ratio and total number of decoded packets.

**Algorithm**	**PCR ± std**	**Decoded packets**
**Realistic uniformly spaced vehicles**		
SSFA	0.1115 ± 0.0886	6,706,167
NORAC	0.1530 ± 0.1040	6,851,859
FABRIC	0.1144 ± 0.0902	6,736,288
**Realistic urban scenario**		
SSFA	0.1341 ± 0.0620	1,368,140
NORAC	0.1344 ± 0.0619	1,286,087
FABRIC	0.1536 ± 0.0649	1,457,903

### Realistic urban scenario

**Figure 5:**
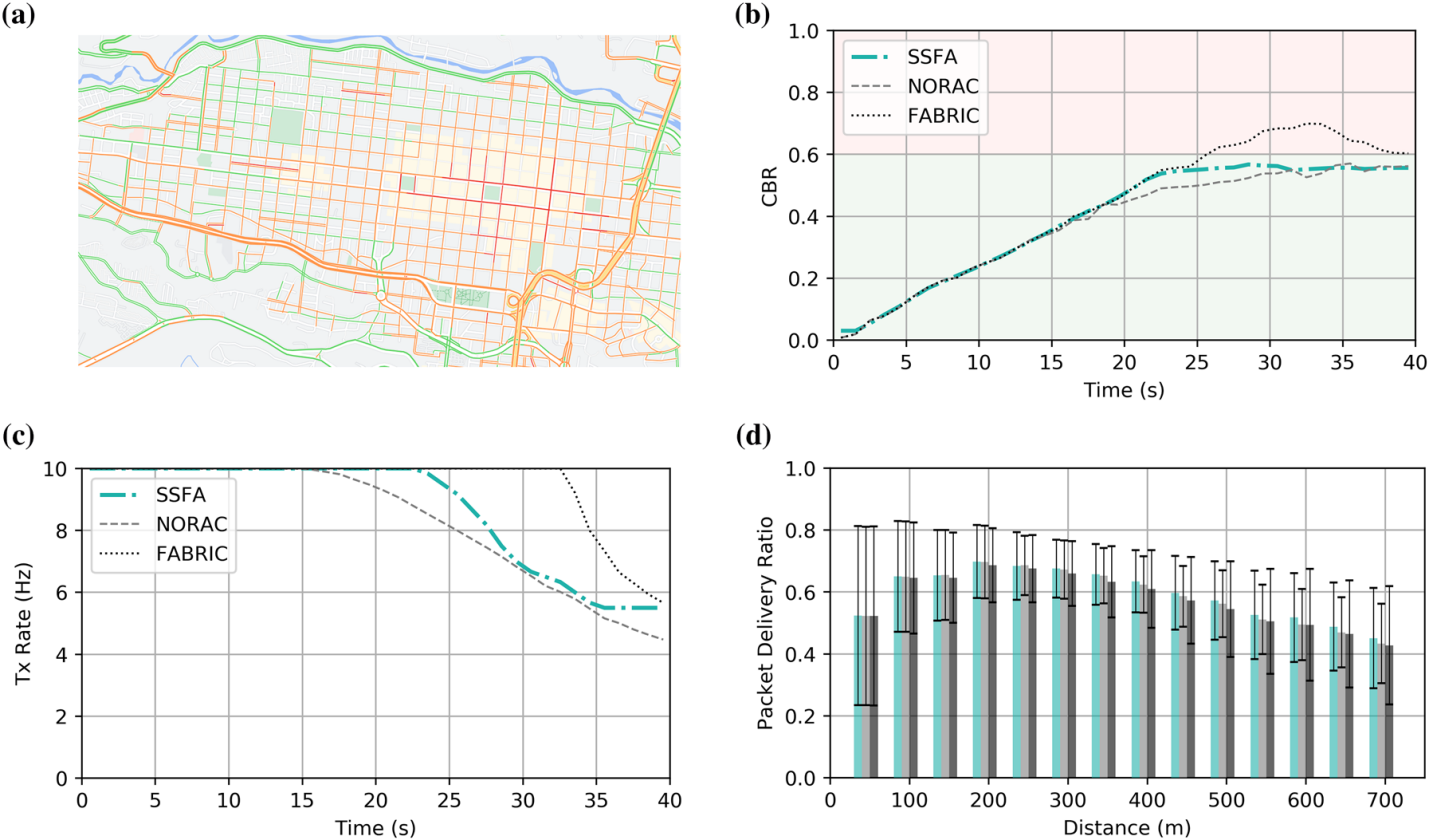
Realistic urban simulation (OMNeT ++ and SUMO) of the proposed congestion control approach compared to FABRIC and NORAC. (**a**) Traffic map (Map data ©2021 Google) of the city of Pereira (Risaralda, Colombia), used in the simulations, illustrating different levels of congestion (from low; green, to high; red) during the peak period (4 p.m.); (**b**) CBR measured and (**c**) allocated beaconing rate of a sample vehicle over time; (**d**) average packet delivery ratio for different vehicles over distance.

Finally, we evaluate SSFA in a realistic urban scenario where, unlike in the previous subsections, vehicles are not uniformly spaced. This will put the non-cooperative scheme to the test because the requirements of the vehicles might differ significantly among neighboring vehicles. For instance, some vehicles could experience congestion while stopped in a traffic jam or at a traffic light, whereas others could flow at higher speeds in a secondary street. In short, this realistic scenario will show how well SSFA performs under congested and stressful conditions considering rapid variations. To this end, in addition to OMNeT +  + and INET, we use Simulation of Urban MObility (SUMO)^44^, a traffic simulation package designed for large networks. The OSM web wizard of SUMO allowed us to select a geographic region and specify traffic mode and demand. We imported the traffic map of the city of Pereira, Colombia. The different levels of congestion in this city (from low; green, to high; red) during the peak period (4 p.m.) are illustrated in Fig. 5a. To simulate this congestion, we sequentially introduce up to 400 vehicles, which will be randomly traveling around the city during the whole simulation time (40 s). The behavior of the first vehicle introduced in the network will be studied so that the congestion experienced by this vehicle increases over time. The goal of this experiment is to observe whether the SSFA approach performs well in terms of convergence time as well as to obtain an adequate CBR compared to other solutions. As can be shown in Fig. 5b, SSFA and NORAC cause the CBR to be the set MBR. However, FABRIC maintains the maximum beaconing rate even after experiencing congestion (Fig. 5c) so the CBR takes longer to converge and to reach the target MBR. This is a disadvantage of cooperative schemes under varying conditions. Concerning the PDR (Fig. 5d), SSFA obtains higher values than its counterparts in almost every distance measured, from 0 to 700 m. It should be noted that the location of vehicles is now random around the simulated urban environment so the PDR has a different distribution than in the previous scenario. The PCR obtained, shown in Table 3, is also similar to or lower than that of FABRIC and NORAC. Therefore, not limiting the CBR properly may lead the algorithms to transmit pointless beacons that not only will be lost but that will impede the reception of DENM messages. In other words, we want to transmit only those messages that will be successfully decoded (Table 3) in order to not congest the channel. Finally, note that vehicles are now introduced gradually so that the total number of decoded packets is smaller with respect to the previous scenario.

## Conclusions and future work

V2V communications are based on transmitting periodic messages (beacons) which support most safety applications and driver assistance systems. However, the associated channel load stemming from beacons should be controlled since it might saturate the channel and hamper the appropriate operation of these applications and services. For this reason, congestion control algorithms aimed at maintaining a given fraction of the channel free are of great importance to preserve the safety of road users, especially by guaranteeing the delivery of emergency-related notifications (DENMs). In this work, we introduce innovative beaconing rate control to alleviate congestion. We make use of approximate reinforcement learning, which allows vehicles to take sensible actions with low computational cost and converge in a short period of time. Our proposal, called SSFA, restricts the channel load by adapting the beaconing rate in a non-cooperative way. Since no additional information from neighbors is required and vehicles work independently, the algorithm is robust even in unfavorable conditions in which packet losses are significant. Moreover, SSFA operates in a distributed manner, thus no pre-installed infrastructure is required for its operation. Results reveal that SSFA successfully maintains channel usage at the desired level, leaving channel capacity free enough for successful DENM reception. Also, a higher packet delivery ratio and a lower number of collisions than other related mechanisms are achieved. In future works, we will focus on the design of algorithms with improved learning capabilities while driving in real implementations.
